# *Retrovirology *highlights a quarter century of HTLV-I research

**DOI:** 10.1186/1742-4690-2-15

**Published:** 2005-03-02

**Authors:** Kuan-Teh Jeang

**Affiliations:** 1 Laboratory of Molecular Microbiology, NIAID, NIH Bethesda, Maryland 20892, USA

## Abstract

In 1977, Takatsuki and co-workers described in Japan a human malignant disease termed adult T-cell leukemia (ATL). Three years later, in 1980, Gallo and colleagues reported the identification of the first human retrovirus, human T-cell leukemia virus type I (HTLV-I), in a patient with cutaneous T-cell lymphoma. This month, *Retrovirology *commemorates these two land mark findings by publishing separate personal recollections by Takatsuki and Gallo respectively on the discovery of ATL and HTLV.

*Retrovirology *as a medical study first emerged in the early 1900s. In 1908, Ellermann and Bang reported on the transmissibility of avian leucosis by cell-free filtrates, suggesting the involvement of a virus [1]. Shortly afterward, in 1910, Rous demonstrated that chicken sarcomas were infectious and when inoculated into healthy birds induced tumors [2]. Today, a plethora of oncogenic animal retroviruses including bovine leukemia virus, feline leukemia virus, gibbon ape leukemia virus, Jaagsiektse sheep retrovirus, murine leukemia virus, mouse mammary tumor virus, reticuloendotheliosis virus, simian T-cell lymphotropic virus, and Walleye dermal sarcoma virus has been described.

Understanding how retroviruses cause cancer took a major step forward with the development of the cellular oncogene hypothesis in 1976. Thus Varmus, Bishop and colleagues [3] demonstrated that the viral oncogenes (*v-onc*) encoded by many retroviruses were captured originally from cellular sequences (i.e. *c-onc*). To date, three general models of retroviral transformation are accepted: a) over-expression of *v-onc*; b) *cis*-oncogenic effect from promoter insertion; and c) *cis*-oncogenic effect from enhancer insertion (Fig. 1A, B, C).

**Figure 1:**
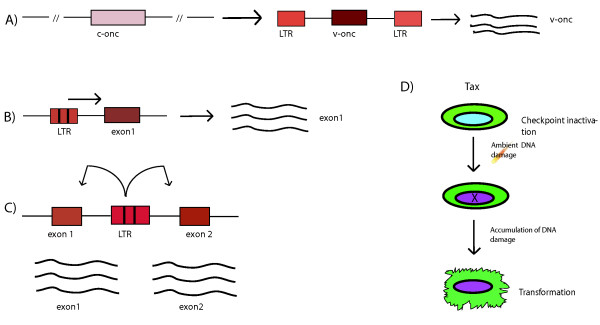
Panels A, B, and C show the three accepted ways by which a retrovirus may transform cells: capture of a *c-onc *and over-expression of *v-onc *by the provirus (A); promoter insertion upstream of a growth controlling cellular gene (B); and enhancer insertions either upstream or downstream of growth controlling cellular genes (C). Panel D shows the stepwise ways in which HTLV-I Tax oncoprotein may transform cells by i) inactivating checkpoints to induce tolerance of damaged DNA, and ii) permitting the accumulation of unrepaired DNA lesions which ultimately convert a normal cell to a transformed cell.

Although not yet fully understood, HTLV-I is believed to transform human T-cells neither through the acquisition of a *c-onc *nor by *cis*-insertion effects on the cellular genome. Pioneering molecular biology studies by Mitsuaki Yoshida and colleagues led to the delineation of the HTLV-I transforming gene, *Tax *[4]. Tax has no cellular homologue; and it works in *trans *to disrupt cellular checkpoints and destabilize genome integrity [5] leading to transformation (Fig. 1D). A more extensive discussion of the molecular biology of HTLV-I and its transforming function will be in an upcoming comprehensive review by Masao Matsuoka to be published in *Retrovirology*.

Two articles in this month's Retrovirology describe respectively the discovery of adult T-cell leukemia [6] and HTLV-I [7].
